# Predictors of changes in cerebral perfusion and oxygenation during obstructive sleep apnea

**DOI:** 10.1038/s41598-021-02829-4

**Published:** 2021-12-06

**Authors:** Zhongxing Zhang, Ming Qi, Gordana Hügli, Ramin Khatami

**Affiliations:** 1 Center for Sleep Medicine, Sleep Research and Epileptology, Clinic Barmelweid AG, 5017 Barmelweid, Switzerland; 2 Barmelweid Academy, Clinic Barmelweid AG, Barmelweid, Switzerland; 3 Department of Neurology, Inselspital, Bern University Hospital and University of Bern, Bern, Switzerland

**Keywords:** Sleep disorders, Near-infrared spectroscopy

## Abstract

Obstructive sleep apnea syndrome (OSAS) is a common sleep disorder. Severe OSAS defined as apnea–hypopnea index (AHI) ≥ 30/h is a risk factor for developing cerebro-cardiovascular diseases. The mechanisms of how repetitive sleep apneas/hypopneas damage cerebral hemodynamics are still not well understood. In this study, changes in blood volume (BV) and oxygen saturation (StO2) in the left forehead of 29 newly diagnosed severe OSAS patients were measured by frequency-domain near-infrared spectroscopy during an incremental continuous positive airway pressure (CPAP) titration protocol together with polysomnography. The coefficients of variation of BV (CV-BV) and the decreases of StO2 (de-StO2) of more than 2000 respiratory events were predicted using linear mixed-effect models, respectively. We found that longer events and apneas rather than hypopneas induce larger changes in CV-BV and stronger cerebral desaturation. Respiratory events occurring during higher baseline StO2 before their onsets, during rapid-eye-movement sleep and those associated with higher heart rate induce smaller changes in CV-BV and de-StO2. The stepwise increased CPAP pressures can attenuate these changes. These results suggest that in severe OSAS the length and the type of respiratory event rather than widely used AHI may be better parameters to indicate the severity of cerebral hemodynamic changes.

## Introduction

Obstructive sleep apnea syndrome (OSAS) is a common sleep disorder with a prevalence of 9–38% in the general population^1,2^. Currently, the severity of OSAS is classified by the apnea–hypopnea index (AHI), which is the number of apnea and hypopnea events per hour during sleep^3^: 5 ≤ AHI < 15 is mild, 15 ≤ AHI < 30 is moderate and AHI ≥ 30 is severe. Epidemiological studies have repeatedly confirmed that the severity of OSAS plays a significant role in the development of cerebrovascular diseases including stroke^4–7^, although the cut-off values of AHI are different in these studies. For example, Redline et al. reported that OSAS patients with AHI ≥ 20 in men and ≥ 25 in women have significantly increased risk of stroke compared with those without OSAS (i.e., AHI < 5)^4^. The same cut-off of AHI ≥ 20 associated with increased risk of stoke is also founded by Arzt et al.^5^ whereas cut-off values given by Yaggi et al.^6^ and Bassetti et al.^7^ are AHI ≥ 35 and ≥ 30, respectively.

A better understanding of how recurrent sleep apneas/hypopneas affect cerebral autoregulation (CA) may provide valuable insights into the acute and chronic consequences of OSAS, including cerebral damage^8–11^. Intact CA during both wake and sleep is essential to maintain a sufficient perfusion of brain tissue and a prerequisite to prevent acute and chronic brain damage^12–14^. Accumulating data in the literature, including human data assessed in vivo, have demonstrated mechanisms of brain damage and subsequent neuronal dysfunctions caused by respiratory events in patients with OSAS. For example, several studies demonstrated reductions in cortical grey matter and changes in hippocampal structures in patients with OSAS using voxel based morphometry (VBM)^15–17^, magnetic resonance imaging (MRI)^17,18^ and spectroscopy (MRS)^19^. Previous studies also reported impaired cerebrovascular regulation in OSAS using MRI arterial spin labeling (ASL)^20^, near-infrared spectroscopy (NIRS)^8,21–23^ and transcranial Doppler (TCD)^10,11,13^, suggesting that OSAS patients chronically exposed to high frequent (i.e., high AHI) repetitive hypoxemia and cerebral perfusion changes are at permanent risk to develop cerebral ischemia due to recurrent hypopnea/apnea events. Indeed, a recent study demonstrated recurrence of cerebral deoxygenation along with recurrence of previously treated nocturnal breathing disturbances when therapeutic continuous positive airway pressure (CPAP) was withdrawn^22^. While these studies confirmed the repetitive changes in cerebral deoxygenation and perfusion directly caused by the respiratory events, the underlying mechanisms determining the severity of the changes are still unknown.

Change in cerebral hemodynamics is a net effect resulting from a combination of multiple pathophysiological factors that challenge CA in OSAS, e.g., cerebral deoxygenation, cardiac output and heart rate (HR), intrathoracic pressure (ITP) swings, hypercapnia, hypoxia, sleep stages and arousals. For example, the ITP swings during OSAS events are associated with decreased cardiac output and HR^24–26^, leading to a reduced global blood/oxygen supply to the brain. On the other hand, acute hypoxia and hypercapnia cause an increase in cerebral blood flow (CBF) via dilations of cerebral arteries and arterioles^27^, counteracting the increase in blood pressure and blood supply. In addition, sleep related factors such as different sleep stages^28–30^, and arousals can also cause changes in cerebral hemodynamics^31,32^, adding to the complexity of cerebral regulation during sleep.

In this study we aim to investigate how the aforementioned different pathophysiological mechanisms quantitatively affect changes in cerebral perfusion and oxygenation in patients with severe OSAS, i.e., a cohort that is at high risk of cerebrovascular diseases, using a stepwise incremental CPAP titration protocol: 1-h sleep without CPAP served as the self-controlled baseline followed by stepwise increment of 1-cmH2O pressure per-hour starting from 5 to 8 cmH2O depending on the individuals. We used NIRS, a well-validated optical technique in assessing the cerebral hemodynamic changes in apneas/hypopneas^8,14,21–23,33,34^, to measure the local hemodynamic changes induced by apneas/hypopneas in microvascular bed which is more challenging for CA^35,36^. NIRS is sensitive to hemodynamic changes in microvascular bed (i.e., arterioles, venules and capillaries)^37,38^. It can simultaneously measure changes in both blood volume (BV) and tissue oxygen saturation (StO2), thus providing better insights into the cerebral hemodynamic consequences of OSAS. We hypothesize that AHI may not be the only factor determining the degree of cerebral hemodynamic changes induced by apneas and hypopneas considering the aforementioned complexity of cerebral hemodynamic regulations; but it may be the key factor considering that it currently defines the severity of OSAS.

## Results

### Changing trends of cerebral hemodynamics

**Figure 1:**
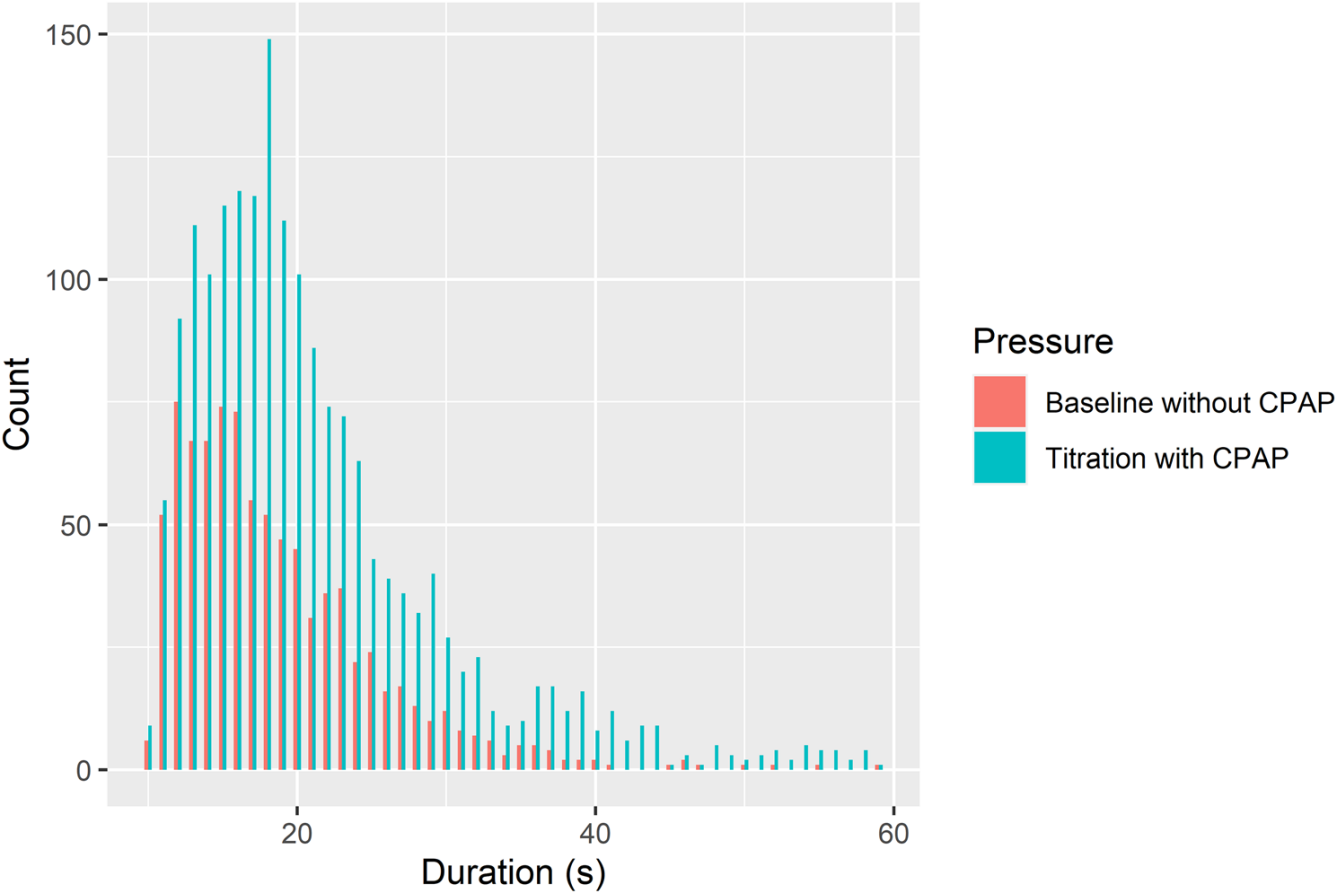
The histogram of the durations of the respiratory events under the measurements without CPAP (n = 884) and with the CPAP (n = 1873). 55 events longer than 60 s during titration are not shown.

In total our frequency-domain multi-distance (FDMD)-NIRS system successfully measured 884 events (i.e., 205 obstructive apneas and 679 hypopneas) at 1-h baseline recordings without CPAP and 1873 events (i.e., 239 obstructive apneas and 1634 hypopneas) during stepwise CPAP titrations in 29 newly diagnosed severe OSAS patients. The results of PSG recordings are listed in Table 1. The distributions of the durations of the events at baseline and during titration are shown in Fig. 1. The most frequent events were 12–16 s at baseline and 13–20 s during CPAP titration.

**Table 1:** The demographics of patients and results of polysomnography.

	1-h baseline sleep	CPAP titration sleep
Age (years)	54.7 ± 2.6	
Sex: male/female	26/3	
BMI (kg/m^2^)	35.8 ± 1.4	
Sleep latency (min)	13.6 ± 2.7	
Minimal CPAP pressure (cmH_2_O)	0	6.8 ± 0.2
Maximal CPAP pressure (cmH_2_O)	0	13.4 ± 0.3
Total recording time (min)	59.2 ± 1.4	412.8 ± 9.0
Total sleep time (min)	38.2 ± 3.3	315.3 ± 16.5
Sleep efficiency (%)	64.0 ± 5.2	76.3 ± 3.6
Number of awakenings	4.4 ± 1.0	39.6 ± 5.5
Wake after sleep onset (min)	5.9 ± 1.4	90.5 ± 14.9
AHI (/h)	70.0 ± 8.1	32.9 ± 5.0
Oxygen desaturation index (/h)	69.3 ± 6.9	28.0 ± 4.2
Limb movement index (/h)	77.8 ± 8.6	76.5 ± 12.7
Arousal index (/h)	56.8 ± 6.7	28.6 ± 2.9
Mean SpO2 (%)	91.4 ± 0.3	92.8 ± 0.3
Lowest SpO2 (%)	84.8 ± 0.8	84.1 ± 1.3

Continuous positive airway pressure (CPAP). Body mass index (BMI). Apnea–hypopnea index (AHI). Data are expressed as the mean ± standard error.

**Figure 2:**
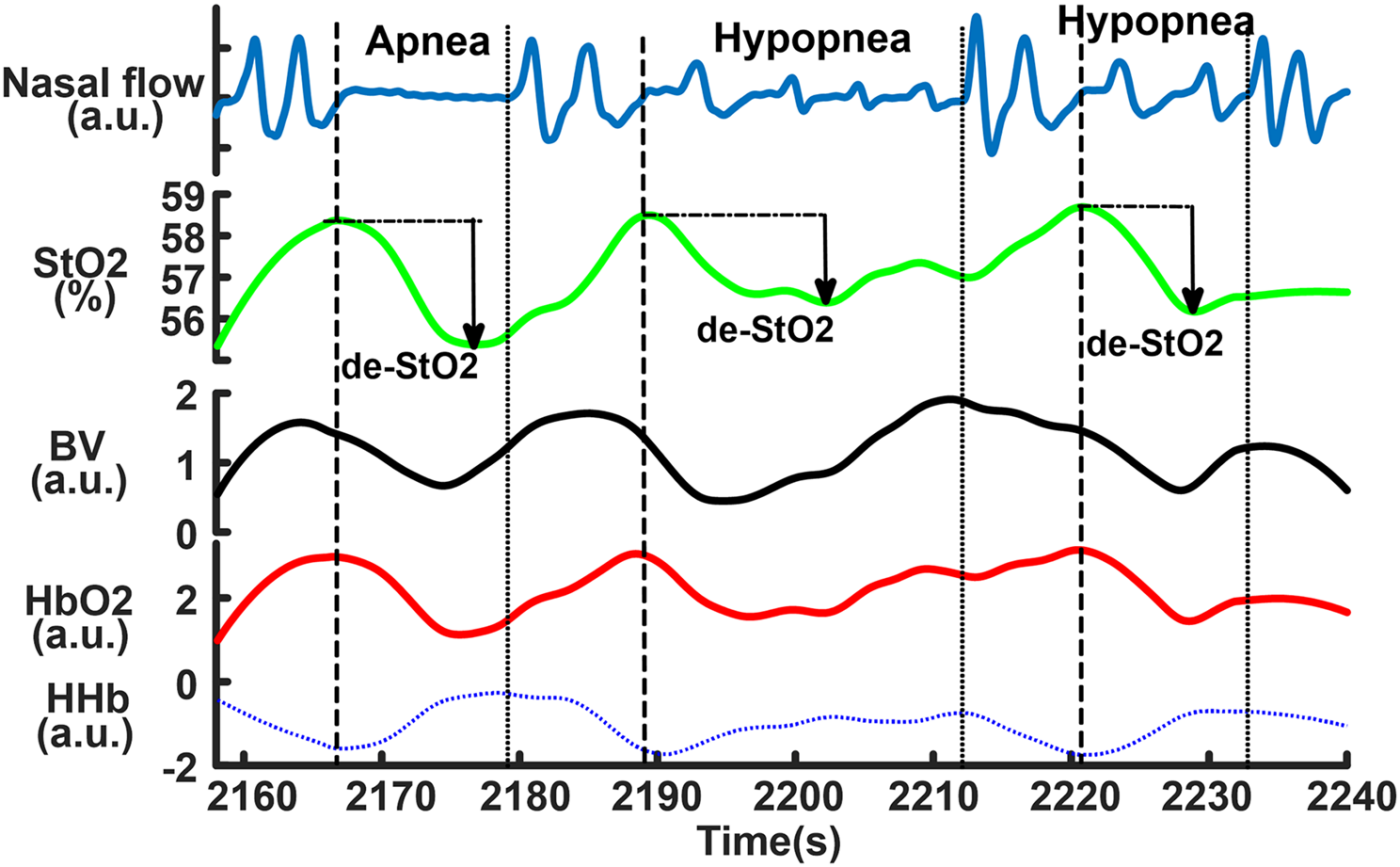
The samples of near-infrared spectroscopy (NIRS) changes in obstructive sleep apneas and hypopneas. The dash lines indicate the start and the end of the events. The cerebral desaturations (de-StO2) are marked in the three events. BV is blood volume. a.u. is arbitrary unit.

Figure 2 shows a sample of NIRS changes. Cerebral oxygenated hemoglobin (HbO2) and StO2 decreased and deoxygenated hemoglobin (HHb) increased after the onsets of the events and their changes were reversed after the end of the events, which was a typical hemodynamic pattern triggered by obstructive apneas and hypopneas reported in previous studies^8,21,23,34^. There was a phase shift between BV and StO2. BV started to decrease before the event onsets and then increased after reaching its nadir within the events, while StO2 started to decrease after the event onsets and it reached its nadir later than BV. The increased BV within the events could be due to cerebral vasodilation triggered by acute hypoxia and hypercapnia. The decrease in BV that occurred after the end of the events could indicate cerebral vasoconstriction in the recovery phase.

### Predictors of cerebral hemodynamic changes determined by LMM

**Figure 3:**
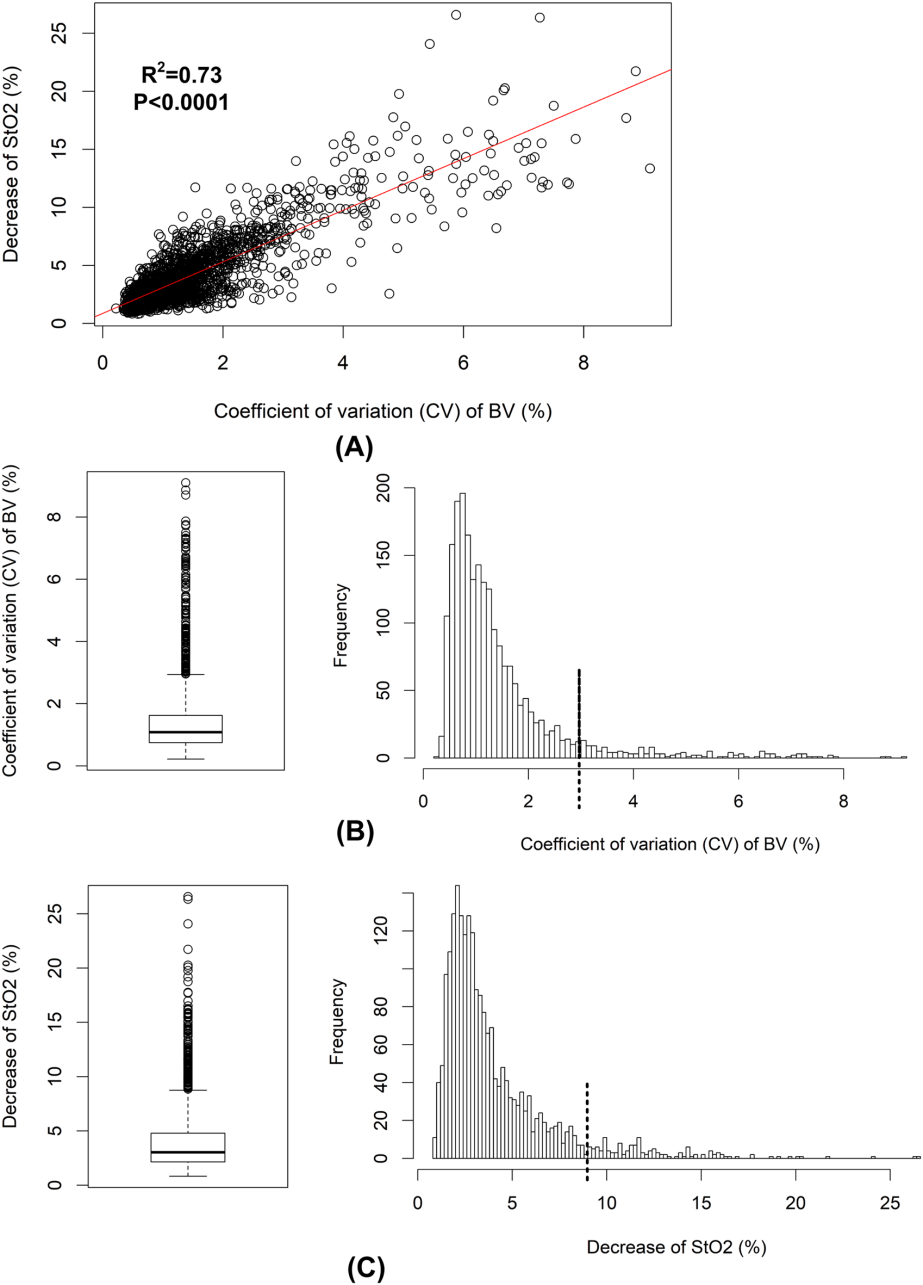
The distributions of the coefficient of variation (CV) of cerebral blood volume (BV) and the decrease of cerebral StO2 (de-StO2). Changes in CV of BV and de-StO2 are correlated as shown in (**A**). The box plot suggests the outliers of CV of BV are larger than 3% and the cut-off 3% is marked with the dash line in its distribution (**B**). Similarly, the cut-off for outliers is 8.7% as shown in the box plot of de-StO2 and it is marked with the dash line in the distribution (**C**).

The coefficient of variation (CV) of BV (CV-BV) and the decreases of StO2 (de-StO2) correlated significantly as shown in Fig. 3A. The distribution of the CV-BV is shown in Fig. 3B and its mean value was 1.41 ± 0.02% (median: 1.09%, interquartile range [IQR]: 0.74–1.62%). Its maximum was 3% as shown in the box plot in Fig. 3B, meaning data larger than 3% were outliers (n = 156). Similarly, the distribution of the de-StO2 (mean value: 4.02 ± 0.06%, median: 3.03%, IQR: 2.15–4.79%) is illustrated in Fig. 3C and box plot suggested that data larger than 8.7% were outliers (n = 149). Thus, 2011 (i.e., 247 obstructive apneas and 1764 hypopneas) and 2018 (i.e., 248 obstructive apneas and 1770 hypopneas) events were finally used to build the LMMs to predict the CV-BV and the de-StO2, respectively. The explanatory variables were age, sex, body-mass-index (BMI), sleep stages, sleep positions, type and duration of respiratory event, mean HR during events, the baseline StO2 before the onset of the event, CPAP pressures and the per-hour AHI, arousal-index (AI) and leg-movement-index (LMI) under each CPAP pressure.

The outcomes of the final LMMs selected by stepwise regressions are shown in Tables 2 and 3. The conditional *R*^*2*^ and *Ω*^*2*^ of the model for CV-BV were 0.77 and 0.65, respectively. These two values of the model for de-StO2 were 0.81 and 0.72, respectively. In both models age, sex, BMI, AHI at diagnosis, hourly AHI and LMI were eliminated by stepwise selections. The results can be summarized as:

1. The duration and type of the events were the most significant covariates determining the changes in cerebral hemodynamics. The changes in cerebral perfusion (i.e., CV-BV) and cerebral desaturation (i.e., de-StO2) were larger in the events of longer durations after the other covariates were controlled for. Obstructive apneas induced larger CV-BV and cerebral desaturation compared to hypopneas.
2. Events associated with larger mean HR and higher baseline StO2 induced smaller changes in cerebral perfusion and desaturation.
3. Events in rapid eye movement (REM) sleep caused smaller changes in CV-BV and de-StO2 compared to the ones in non-REM (NREM) sleep.
4. Arousal system influenced the induced changes in cerebral perfusion but not in cerebral desaturation.
5. Sleep position did not affect cerebral desaturation but only the perfusion. The events caused larger changes in CV-BV when the patients slept on their right side or on their back compared to sleep on the left side. There was no difference in CV-BV between sleeping on the right side and in supine position (stepwise regression gave t-value = 1.60, P-value = 0.11).
6. Finally, increased CPAP pressure can stepwise attenuate the event-related changes in cerebral hemodynamics.

**Table 2:** The outcomes of the linear mixed-effects model of the CV-BV changes.

	Estimate (10^–3^)	95% CI (10^–3^)	t-value	P-value
Duration of events	2.66	[1.54, 3.78]	4.67	< 0.0001
Apnea–hypopnea	195.3	[138.52, 252.08]	6.74	< 0.0001
Mean HR within events	− 5.16	[− 7.55, − 2.77]	− 4.24	< 0.0001
Hourly arousal index	2.14	[1.26, 3.02]	4.8	< 0.0001
Baseline StO2	− 10.77	[− 17.08, − 4.46]	− 3.34	0.00085
REM sleep–NREM sleep	− 64.75	[− 123.45, − 6.05]	− 2.16	0.031
**Sleep positions**				
Right side	172.5	[104.59, 240.41]	4.98	< 0.0001
Supine	130.0	[68.93, 191.07]	4.17	< 0.0001
CPAP pressures	− 5.93	[− 10.91, − 0.95]	− 2.33	0.0198

Confidence interval (CI). Heart rate (HR). Rapid eye movement sleep (REM). Non-REM sleep (NREM). Continuous positive airway pressure (CPAP). The change in apnea is the reference for the change in hypopnea in this model. Sleep on left side is the reference for sleep on right side and on supine position. NREM sleep is the reference for REM sleep. Hourly arousal index is the arousal index calculated in each hour.

**Table 3:** The outcomes of the linear mixed-effects model of the decrease of StO2.

	Estimate (10^–2^)	95% CI (10^–2^)	t-value	P-value
Duration of events	1.96	[1.65, 2.27]	12.26	< 0.0001
Apnea–hypopnea	24.34	[9.64, 39.04]	3.25	0.0012
Mean HR within events	− 0.96	[− 1.61, − 0.31]	− 2.95	0.0032
Baseline StO2	− 5.30	[− 6.85, − 3.75]	− 6.71	< 0.0001
REM sleep–NREM sleep	− 24.04	[− 39.39, − 8.69]	− 3.07	0.0022
CPAP pressures	− 1.55	[− 2.55, − 0.55]	− 3.03	0.0025

Confidence interval (CI). Heart rate (HR). Rapid eye movement sleep (REM). Non-REM sleep (NREM). Continuous positive airway pressure (CPAP). The change in apnea is the reference for the change in hypopnea in this model. NREM sleep is the reference for REM sleep.

**Figure 4:**
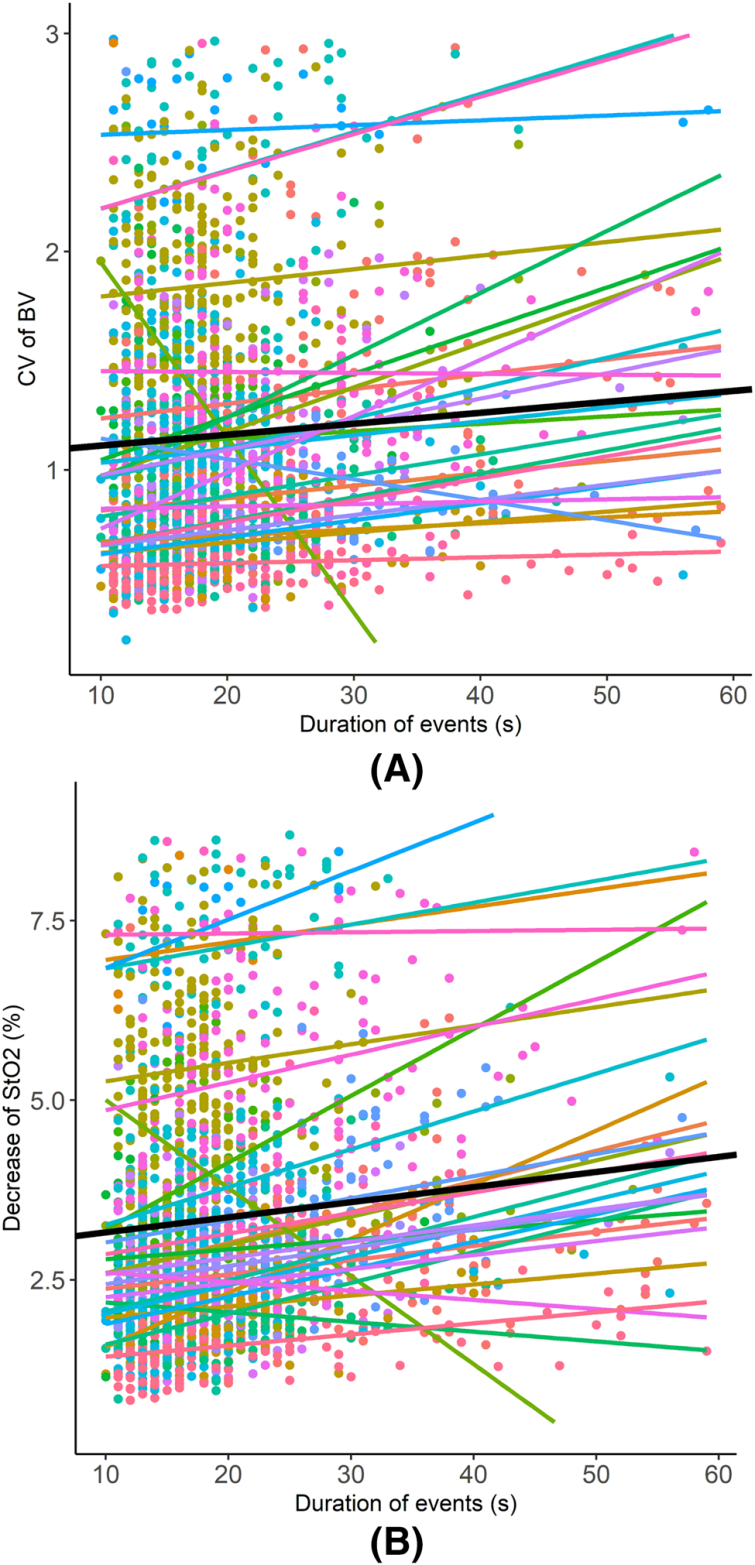
The changes in the coefficient of variation (CV) of cerebral blood volume (BV) and the decrease of cerebral tissue oxygen saturation (StO2) versus the duration of events in each patient. The data points of different colors indicate data from different patients, and the lines of different colors are the linear fitting of the data points. The black line is the mean of all patients. We only show the events of durations shorter than 60 s, considering that only a minority of events is longer than 60 s and they may be outliers that can bias the fitting trends.

Since we did not expect to find that the event’s length rather than AHI was the most significant predictor of the changes in cerebral hemodynamics, we illustrate the changes in CV-BV and de-StO2 versus the event’s duration in each patient in Fig. 4. The individual linear fitting lines in Fig. 4 suggest that indeed in the majority of the patients the changes in CV-BV and de-StO2 increased with increased duration.

## Discussion

Our study for the first time quantifies the relative effect sizes of different pathophysiological mechanisms contributing to the cerebral hemodynamic changes in local microvascular bed induced by obstructive sleep apneas and hypopneas. The strengths of our study are: (1) we use a stepwise incremental CPAP titration protocol in which the first hour of sleep without CPAP served as a self-control in newly diagnosed untreated patients. The stepwise CPAP titration gradually opens the upper airway, increases ITP, reduces hypoxia and hypercapnia, reduces the number of apneas/hypopneas and arousals, and finally consolidates sleep. This protocol allows us to gradually control and identify the most relevant factors from many potential physiological factors in a systematic way, and our results are not affected by pre-treatment effects, as our patients were newly diagnosed; (2) we simultaneously quantify the changes in both cerebral perfusion (i.e., changes in BV) and oxygenation (i.e., changes in StO2), thus providing better insights into the relationship between local cerebral metabolism and blood supply during apneas/hypopneas. Remarkably, we find that duration and type of respiratory event rather than AHI are the most significant covariates determining the cerebral hemodynamic changes in severe OSAS. Longer events induce stronger changes in cerebral perfusion and larger cerebral desaturation. Obstructive apneas lead to larger changes in cerebral BV (CBV) and desaturation than hypopneas. In addition, brain activity (sleep stages, arousal and baseline cerebral oxygenation) as well as systemic parameter (i.e., HR and CPAP pressures) affect cerebral hemodynamics. Finally, our data demonstrate that CPAP is able to attenuate the cerebral hemodynamic changes triggered by the apneas/hypopneas.

Given that the event’s inherent features such as duration and the type of event are the most significant variables after controlling for other covariates (these results are also valid in naive apneas/hypopneas at baseline, see “Supplemental Results”), our findings have important scientific and clinical implications. Our results suggest that the duration and type of the event rather than AHI is a useful parameter indicating the severity of the altered cerebral hemodynamics in severe OSAS. Epidemiological studies investigating the cerebro-cardiovascular risks of OSAS should consider the length and type of respiratory events as predictors in the future. In clinical practice, a summary of the length of respiratory events may be needed in traditional PSG report in addition to AHI. Patients with extremely long apneas/hypopneas may require special attention, and CPAP treatment should be highly recommended even if their AHI values are not high because a progressive increase in the events’ lengths may induce a powerful challenge to cerebrovascular autoregulation and an increase in cerebrovascular risk. Our models also provide quantitative tools to assess the severity of cerebral hemodynamic changes. According to our model, a duration longer than 5 s could induce 1.33 × 10^–2^ stronger (i.e., 2.66 × 10^–3^ × 5) changes in CV-BV but the obstructive apnea in general causes 19.53 × 10^–2^ more changes than hypopnea. Thus, a 15-s hypopnea will cause larger changes in cerebral perfusion than a 10-s hypopnea but smaller changes in cerebral perfusion compared to a 10-s obstructive apnea.

The contributions of different covariates to the cerebral hemodynamic regulations quantified by our models are reasonable and interpretable and are consistent with each other. Higher HR during the apnea/hypopnea events may indicate relatively higher sympathetic activation and less reduction in systemic blood supply^25,39^. Greater systemic blood supply is available for the brain in these events compared to in those with lower HR; thus, they trigger smaller changes in cerebral perfusion and StO2. This could consistently explain why we found that higher baseline StO2 before the onsets of events could prevent the subsequent changes in cerebral perfusion and desaturation. Previous studies found that longer apnea events are associated with a greater decrease in HR (i.e., lower HR during the events)^39^. Therefore, it is reasonable that in our model the duration of the event and the mean HR within the event have contrary influences to the cerebral hemodynamics (see their fitted coefficients in Tables 2 and 3).

The triggered cerebral hemodynamic changes are smaller in REM sleep compared to in NREM sleep, contrasting with published findings, which show that during nocturnal REM sleep the obstructive apneas/hypopneas are associated with more severe peripheral oxygen desaturations^40–42^. One previous NIRS study also reported that the drops of cerebral StO2 induced by obstructive sleep apneas/hypopneas are larger in REM sleep compared to in NREM sleep^33^. The different results could be explained by: (1) in our stepwise incremental CPAP titration protocol the pressures are higher in REM sleep because REM sleep occurs mainly in the later period of sleep toward morning. Thus, it is more likely that CPAP restores obstructive apneas/hypopneas in REM sleep better compared to in NREM sleep. (2) The previous NIRS study^33^ measured 13 patients during daytime nap without CPAP and only four patients had REM sleep. Most of their patients (8/13) had been on regular CPAP therapy before the study. This suggests that their results are unlikely to be comparable with ours, considering the different study protocols (i.e., nocturnal CPAP titration vs. normal daytime nap), different study populations (i.e., CPAP treatment naïve vs. regular CPAP treatment) and their small number of patients having REM sleep. (3) Cerebral oxygen saturation may be different from the peripheral one, considering the unique cerebral CA mechanism. Previous studies found that the cerebral StO2 measured by NIRS increased continuously over the whole night of sleep^43^, indicating the brain may have higher oxygen supply in REM sleep. Therefore, we may hypothesize that probably even without CPAP the naïve apneas/hypopneas may cause smaller desaturation in the brain in REM sleep compared to in NREM sleep after controlling for the other covariates (i.e., length and type of event, HR, sleep position, etc.), in spite of the stronger desaturation in peripheral tissues. This hypothesis needs to be tested in the future studies.

Sleep position can influence the changes in cerebral perfusion but not cerebral oxygenation. This result can be explained by the gravity that influences local cerebral blood supply and CA^44^. Since our NIRS sensor is placed on the left forehead, higher perfusion in the left hemisphere is measured when the patients sleep on their left side compared to on their right side or on their back. This result indicates that cerebral blood supply can be regulated by factors such as sleep position independent from cerebral oxygen consumption during apneas/hypopneas. The blood volume in skin vasculature could also be influenced by gravity when sleep position changes. However, our results are unlikely to be contaminated by skin vasculature, because we use a FDMD-NIRS technique. Our Imagent system used in this study is currently the only commercial benchtop FDMD-NIRS device^45–47^ and has been CE approved for research. As shown in the “Supplementary file” (“Supplemental Methods”), in our Imagent system the four light sources are aligned and placed at 2 cm, 2.5 cm, 3 cm and 3.5 cm away from the detector, yielding four detection channels with different source-detector distances. A NIRS device using a single source-detector distance cannot distinguish the light absorption from superficial and deeper tissues; thus, skin vasculature can bias the measurement of deeper cerebral hemodynamics. However, in FDMD-NIRS the superficial tissues affect all the four channels similarly; thus, the superficial influences in each channel can be attributed to the intercepts and the residuals of the linear regression Eqs. (1–3) in the “Supplemental Methods”. The cerebral hemodynamic parameters measured by FDMD-NIRS are calculated using the slopes of Eqs. (1–3)^46^. In other words, the skin vasculature influence is cancelled out in the calculation of cerebral hemodynamics using the four channels of different source-detector distances. The reliability of our FDMD-NIRS in measuring hemodynamics in deeper tissues such as in the brain has been well validated in previous studies^47–50^.

We do not directly measure CBF but use changes in CV-BV to indicate the changes in cerebral perfusion. Grubb et al. found that the relationship between CBF and CBV in macaque monkeys can be quantified by a simple power law equation that CBV = (CBF)^*G*^^51^. This relation has been confirmed in other animals and humans using various technologies^52^, e.g., when CBV and CBF are both measured by the same technologies such as MRI^53^ and positron emission tomography (PET)^54^, or by different technologies such as when CBV is measured by NIRS and CBF is measured by laser Doppler^55^ and TCD^56^. Although the coefficient *G* that is larger than 0 is different in different studies, the time courses of changes in CBV and CBF are similar, that is, the positively monotonic relationship between CBV and CBF still holds despite non-linear relationship. The formulation of Grubb’s exponent can also be expressed as a proportional change^56,57^, i.e., (CBV/CBV_0_) = (CBF/CBF_0_)^*G*^, where CBV_0_ and CBF_0_ are the baseline values of CBV and CBF. One advantage of this expression is that the units of CBV and CBF are cancelled out as they are normalized to the baseline. As the CV-BV in our study is also a normalized parameter without unit, it is reasonable to treat it as a valid index for the relative changes in CBF thus it can quantify the changes in cerebral perfusion.

## Limitations and perspectives

There are several limitations in this study that need to be further improved in the future studies. First, the AHI is not a significant predictor, suggesting that the frequency of the apnea/hypopnea attacks is no longer relevant for the induced cerebral hemodynamic changes in severe OSAS. This is probably because the CA functions of our patients have been already impaired, considering their age. Whether this is a common phenomenon also existing in mild and moderate OSAS patients is an interesting question needing further experimental studies. Second, the function of CA decreases with aging but age is not a significant predictor for changes in cerebral hemodynamics in our models. One possible explanation is that we only include patients with severe OSAS who need CPAP therapy, and thus we could not include many young patients to cover a larger age range. Third, we only measured the left forehead. Although our results may be generalized to other brain areas because most of the significant predictors in our regression analyses are not specific to the left forehead except for the sleep position, future studies measuring more areas are needed. Finally, we did not include central sleep apneas in the analysis because it is difficult to distinguish the naïve from treatment-emergent central apneas^58^. Future studies scrutinizing the patterns of cerebral hemodynamics with NIRS, including directly measuring CBF with emerging near-infrared diffuse correlation spectroscopy technology^59–61^, may be helpful to classify different central apneas and deepen our knowledge of the pathophysiology of different types of sleep apneas and their hemodynamic consequences to the brain.

## Methods

In total, 29 newly diagnosed OSAS patients (AHI at diagnosis [mean ± standard deviation, std.]: 53.1 ± 24.6/h, IQR: 32.7–70/h) participated in this study. Table 1 shows the demographics of these patients. Patients with unstable coronary or cerebral artery disease, severe arterial hypertension or hypotension, respiratory diseases or a history of a sleep-related accident were excluded. This study was approved by the Kantonale Ethikkommission Aargau, Switzerland, and was in compliance with the declaration of Helsinki.

In the first night, all patients underwent video-polysomnography (PSG) measurement (Embla RemLogic, Natus Medical Incorporated, Tonawanda, NY, USA) for diagnosis. The following day, patients who were diagnosed as OSAS and clinically recommended for CPAP therapy gave their written informed consent. In the following night, patients underwent stepwise CPAP (AirSense™10, ResMed) titration together with video-PSG and NIRS recordings: 1-h sleep without CPAP served as the self-controlled baseline followed by stepwise incremental change of 1-cmH_2_O pressure per-hour starting from 5 to 8 cmH_2_O depending on the individuals.

Video-PSG measured electroencephalography at electrode locations of C3, C4, O1, O2, F3, and F4 according to the 10–20 system, electrooculogram, electromyogram, electrocardiogram, breathing functions, HR, peripheral oxygen saturation and the movement during sleep. Two experienced sleep technologists independently scored the sleep stages, respiratory and limb movement events, and motion artifacts in 30-s epochs according to the 2017 American Academy of Sleep Medicine manual^3^. The discrepancy between these two technologists was solved by their discussion or the recommendation of a third experienced neurophysiologist. The hourly AHI, arousal-index (AI) and leg-movement-index (LMI) under specific CPAP pressure per-hour were also calculated (i.e., the number of events divided by the sleep time under each CPAP pressure per-hour in the titration protocol).

Frequency-domain multi-distance (FDMD)-NIRS (Imagent, ISS, Champaign IL, USA) measurements were conducted over the middle of left forehead. The cerebral HbO2 and HHb were calculated from the fitted slopes of the modulated light amplitude and phase shifts over four various light sources and detector distances^45,46^. The sum of HbO_2_ and HHb can at best be regarded as a metric of BV^45,62,63^. The coefficient of variation (CV) of BV (CV-BV) during apnea/hypopnea and recovery phase was calculated. CV is a standardized measure (i.e., standardization) of the variability of the data in relation to their mean, i.e., CV = 100 × std./mean. It was used in previous NIRS studies quantifying the cerebral hemodynamic changes between subjects as it can eliminate the bias due to individual differences^38,64^. Smaller changes in CV-BV indicated smaller changes in the cerebral perfusion. The absolute value of StO2, i.e., StO2 = 100 × HbO2/(HbO2 + HHb), was calculated in FDMD-NIRS^45^. The mean StO2 at baseline before event onset and the subsequent decrease (de-StO2, which was the maximal value minus the minimal value after event onset) were also calculated. The sample rate of FDMD-NIRS recording was 5.2 Hz. 2-s preceding and 5-s following the sleep apnea/hypopnea event was selected as baseline and recovery phase, respectively. More details of the FDMD-NIRS measurement can be found in the “Supplemental file”.

Linear mixed-effects model (LMM) with a random intercept by patient was used to predict the CV-BV and the de-StO2 caused by the apneas/hypopneas, respectively. The explanatory variables were age, sex, BMI, sleep stages, sleep positions, type and duration of respiratory events, mean HR during event, the baseline StO2 before the onset of the event, CPAP pressures and the per-hour AHI/AI /LMI under each pressure. In each patient, all the events under a specific CPAP pressure were excluded from LMM if the corresponding sleep duration under that pressure was shorter than 20 min to exclude the unreliable calculation of the per-hour AHI/AI/LMI. In total, the data of 2167 events from 27 patients were selected (mean number of events from the patients: 80.3 ± 10.9, median number of events: 71, IQR: 40–99.5). One male was excluded because his position sensor fell off. One female was excluded because of short sleep duration. Box plot was used to graphically depict the CV-BV and the de-StO2. Outliers of the box plots were further excluded from LMM to avoid violating the model assumptions of normality and homoscedasticity. Stepwise regression using backward elimination was done to select the best predictors. We reported both the conditional *R*^*2*^^65^ and *Ω*^*2*^ (i.e., Xu’s *R*^*2*^ calculated as 1 − variance of residual/variance of response)^66^ to assess the goodness of fit of our final selected models.

Data were expressed as the mean ± standard error (SE) unless otherwise indicated. All statistical analyses and modeling were performed using R (version 3.2.4). The LMM models were done using the R package *lme4* and *lmerTest*.

## Supplementary Information

Supplementary Information.

## Data Availability

The data that support the findings of this study are available from the corresponding author upon reasonable request.
