# Supplementary material for: Predictors of changes in cerebral perfusion and oxygenation during obstructive sleep apnea
Source: Sci Rep. 2021 Dec 6;11:23510. doi: 10.1038/s41598-021-02829-4 (PMC8648752; doi:10.1038/s41598-021-02829-4)
Supplement: Supplementary file 1 — Supplementary Information. [file 41598_2021_2829_MOESM1_ESM.docx]

**Supplemental Material**

**Methods**

**Frequency-domain multi-distance (FDMD) near-infrared spectroscopy (NIRS)**

In this study, FDMD-NIRS (Imagent, ISS, Champaign IL, USA) measurements were conducted over the middle of left biceps muscle. Its light emitters, four laser diodes at 690-nm wavelength and four laser diodes at 830-nm wavelength are coupled into four light sources and are high frequency modulated at 110 MHz. The light can penetrate into the measured tissues with a depth of several centimeters when the four light sources are aligned and placed at 2 cm, 2.5 cm, 3 cm and 3.5 cm away from an optical fiber bundle connected to the photomultiplier tube detector. As illustrated in Fig.S1, in FDMD-NIRS the light emitted from light source can be detected by detectors placed at different distances away from the source. The light intensity ($I\text{DC}$) and modulation amplitude ($\text{I}\text{AC}$) of the detected light decrease and phase delay ( $\phi$ ) occurs between the detected light and the source light due to the absorption and scattering. The detected $I\text{DC}$ and $\text{I}\text{AC}$ are smaller but the $\phi$ is larger at the detector further away from the light source. The $I\text{DC}$, $\text{I}\text{AC}$ and $\phi$ from different light source-detector distances actually vary linearly [1, 2]. Therefore, to submit the measured$\text{I}\text{DC}$, $I\text{AC}$ and $\phi$ to linear regression we can obtain the following equations derived from photon diffusion equation in a semi-infinite geometry [1, 3-6]:

$ln\text{(}r^{2}\text{I}\text{AC}\text{)=}r\text{S}\text{AC}\text{ +}\text{C}\text{AC}$ (1)

$ln\text{(}r^{2}\text{I}\text{DC}\text{)=}r\text{S}\text{DC}\text{ +}\text{C}\text{DC}$ (2)

$\phi=rS_{\phi}\text{ +}C_{\phi}$ (3)

Where $r$ is the known source-detector distance, $S\text{AC}$, $S\text{DC}$ and $S_{\phi}$are the slopes and $C\text{AC}$, $C\text{DC}$, $C_{\phi}$ are the intercepts. Combing any two of these three slopes (e.g., we chose$\text{S}\text{AC}$ and $S_{\phi}$ in the following equations) we can calculate $\mu_{a}$and $\mu_{s}'$of the measured tissue [1, 3-6]:

$\mu_{a}=\frac{\omega}{2\nu}\left( \frac{S_{\phi}}{\text{S}\text{AC}}-\frac{\text{S}\text{AC}}{S_{\phi}} \right)$ (4)

$\mu_{s}' =\frac{{\text{S}\text{AC}}^{2}-{S_{\phi}}^{2}}{3\mu_{a}}-\mu_{a}$ (5)

where $\omega/2\pi$ is the modulation frequency and $\nu$ is the velocity of light in the tissue. FDMD-NIRS uses two wavelengths. $\mu_{a}$ and $\mu_{s}'$of both wavelengths can be calculated individually using the same equations (4) and (5). Equation (4) gives us the absorption coefficient of the measured tissues calculated by taking the influence of scattering into account. In NIRS the main contributions to absorptions in tissues are HbO2 and HHb, so we have:

$\mu_{a}^{\lambda}=ɛ_{HHb}^{\lambda}C_{HHb}+ɛ_{HbO2}^{\lambda}C_{HbO2}$ (6)

where $\mu_{a}^{\lambda}$ is the absorption coefficient of the measured tissue at wavelength λ. $ɛ_{HHb}^{\lambda}$ and $ɛ_{HbO2}^{\lambda}$ are the known extinction coefficients at wavelength λ for HHb and HbO2, respectively. $C_{HHb}$ and $C_{HbO2}$ are the concentrations of HHb and HbO2 respectively. Using two wavelengths λ1 and λ2 we can then calculate $C_{HHb}$ and $C_{HbO2}$ with the following equations:

$C_{HbO2}=\frac{\mu_{a}^{\lambda1}ɛ_{HHb}^{\lambda2}-\mu_{a}^{\lambda2}ɛ_{HHb}^{\lambda1}}{ɛ_{HbO2}^{\lambda1}ɛ_{HHb}^{\lambda2}-ɛ_{HbO2}^{\lambda2}ɛ_{HHb}^{\lambda1}}$ (7)

$C_{HHb}=\frac{\mu_{a}^{\lambda2}ɛ_{HbO2}^{\lambda1}-\mu_{a}^{\lambda1}ɛ_{HbO2}^{\lambda2}}{ɛ_{HbO2}^{\lambda1}ɛ_{HHb}^{\lambda2}-ɛ_{HbO2}^{\lambda2}ɛ_{HHb}^{\lambda1}}$ (8)

Therefore StO2 can be further derived as:

$StO2=100\times\frac{HbO2}{HbO2+HHb}$ (9)

The FDMD-NIRS system is currently well recognized as the most robust and reliable reference commercially available NIRS technique [2, 7, 8], considering its sophisticated mathematical frameworks calculating $\mu_{a}$ and $\mu_{s}'$ that can best estimate the real light propagation distance in the measured tissues based on derivations of the diffusion equation in complex geometries. The robustness, precision and accuracy of measuring HbO2/HHb/StO2 of our Imagent system used in this study have been well validated in different physical blood-lipid models [1, 4, 8] and in vivo studies [9-11].


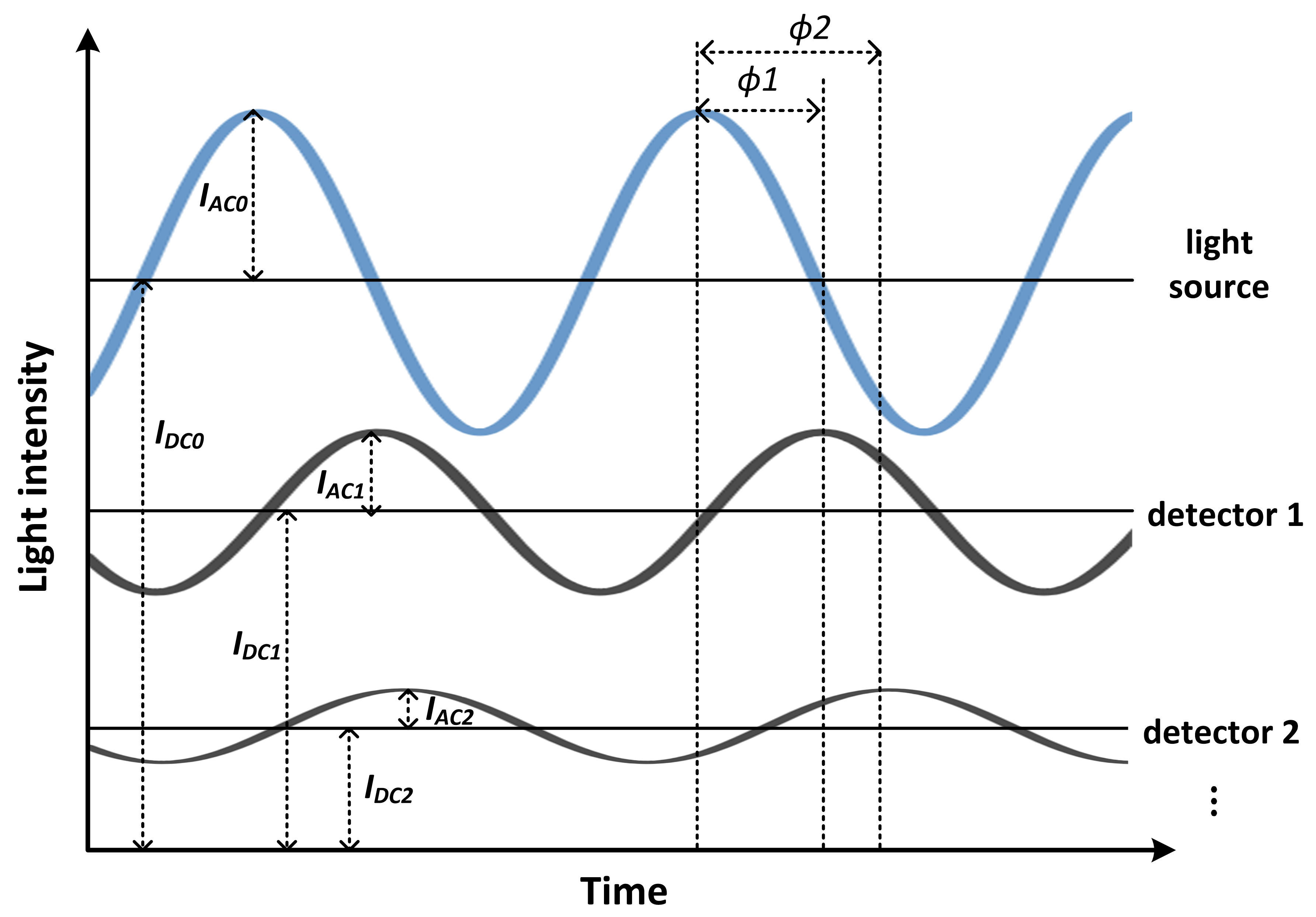


Fig. S1. Frequency-domain multi-distance near-infrared spectroscopy (FDMD-NIRS) measurement. The blue sine wave is the high frequency modulated light source. $I_{DC0}$, $I_{AC0}$ are its light intensity and modulation amplitude. The two black sine waves are the detected output light after passing the measured tissues. They are detected by detectors 1 and 2 placed at different distances away from the light source. The light intensities and modulation amplitudes of the two black sine waves are smaller than the ones of the light source and their phases are delayed because of the absorption and scattering in the tissues. Detector 1 is closer to the light source than detector 2. Therefore, the light intensity $I_{DC1}$ and modulation amplitude $I_{AC1}$ detected at detector 1 are larger than the light intensity $I_{DC2}$ and modulation amplitude $I_{AC2}$ detected at detector 2. The phase delay $\phi1$ at detector 1 is smaller than the phase delay $\phi2$ at detector 2 because the light reaching detector 2 has travelled longer distance in the tissue. Similarly, the light intensity and modulation amplitude will be further decreased and the phase delay will be further increased at the other detectors placed further away than detector 2.

**Pre-processing of FDMD-NIRS signals**

Before the start of every recording in patients, the NIRS device was calibrated on optical phantom blocks. The reliability of FDMD-NIRS measurement depended on the linearity of the raw optical signals on distances, i.e., the linear dependence *R^2^* of modulated light amplitude and phase shift over the measured distances were very similar to 1 in each light wavelength. The raw optical data were discarded if the *R^2^* was smaller than 0.95 in either modulation amplitude or phase shift in any wavelength. This step can exclude poor quality data arising from improper probe-skin contact and shunted light reaching the detectors without travelling through the tissue. The NIRS data were then subjected to a low-pass (<0.08 Hz) zero-phase filter designed using a Hanning window to remove the physiological noise including heart rate (HR), respiratory noise and spontaneous slow hemodynamic oscillations [12, 13]. The filtered data were smoothed with a moving average smoothing method (robust locally weighted scatter plot smoothing [12, 14]). The StO2 values smaller than 30% or larger than 90% were discarded to exclude potential movement artifacts or unreliable recordings, considering that the normal NIRS StO2 baseline value is between 50% and 80% [15, 16], and a drop up to 13% in StO2 is possible in some patients with brain dysfunction [[17]](#_ENREF_40). An entire event was excluded from further analysis if more than 20% of its data were discarded. In each patient, if the mean BV of the event was more than three standard deviations away from the mean BV of all events, then this event was defined as outlier and was excluded from further analysis.

**Results**

**The results of LMM and stepwise regression in native sleep apnea/hypopnea events at baseline without CPAP**

We used the same LMM and stepwise regression approach to further test if the result that the duration of events was the most significant predictor of changes in cerebral perfusion and desaturation in native sleep apnea/hypopnea events (i.e., at baseline without CPAP). The results of the final model for CV-BV and de-StO2 are summarized in Table S1 and Table S2 below, respectively.

1). The duration of the events and the types of events were the most significant predictors in both models after controlling for the other covariates: longer events caused larger changes in cerebral perfusion and desaturation, and obstructive apneas triggered larger changes in cerebral hemodynamics than hypopneas.

2). The mean HR during the events and sleep positions were significant covariates influencing the changes in cerebral perfusion.

3). Baseline StO2 before events was not a significant predictor for changes in cerebral perfusion at baseline, but it was still significant for the cerebral desaturation (Table S2), which probably could be explained by the sleep initiation mechanisms that play an important role in cerebral perfusion in the first 1-hour baseline sleep. Our previous study showed increased cerebral perfusion after sleep onset using NIRS [18], suggesting that cerebral vasodilation may be a universal phenomenon at sleep onset process that is independent from (or less associated with) cerebral StO2.

4). Sleep stages did not influence changes in cerebral hemodynamics at baseline, which could be explained by the fact that in the first hour of sleep these patients rarely have REM sleep.

Table S1. The outcomes of the model of the CV-BV changes at baseline without CPAP.

|  | Estimate | 95% CI | t-value | P-value |
| --- | --- | --- | --- | --- |
| Duration of events | 0.01 | [0.0055, 0.015] | 4.48 | <0.0001 |
| Mean HR within events | -0.0035 | [-0.0064, -0.00056] | -2.27 | 0.024 |
| Apnea-Hypopnea | 0.33 | [0.24, 0.42] | 7.213 | <0.0001 |
| Sleep positions | | | | |
| Right side | 0.14 | [-0.048, 0.33] | 1.48 | 0.13 |
| Supine | 0.289 | [0.13, 0.45] | 3.46 | 0.0006 |

Confidence interval (CI). Heart rate (HR). Sleep on left side is the reference for sleep on right side and on supine position. The conditional *R^2^* and *Ω^2^* of the model for CV-BV were 0.73 and 0.66, respectively.

Table S2. The outcomes of the model of the StO2 changes at baseline without CPAP.

|  | Estimate | 95% CI | t-value | P-value |
| --- | --- | --- | --- | --- |
| Duration of events | 0.043 | [0.032, 0.054] | 8.04 | <0.0001 |
| Baseline StO2 | -0.071 | [-0.11, -0.034] | -3.72 | 0.00022 |
| Apnea-Hypopnea | 0.42 | [0.20, 0.64] | 3.98 | <0.0001 |

The conditional *R^2^* and *Ω^2^* of the model for CV-BV were 0.86 and 0.76, respectively.

**References**

1. Fantini S, Franceschini M-A, Maier JS, Walker SA, Barbieri BB, Gratton E. Frequency-domain multichannel optical detector for noninvasive tissue spectroscopy and oximetry. OPTICE 1995: 34(1): 32-42.

2. Fantini S, Sassaroli A. Frequency-domain techniques for Cerebral and Functional Near-Infrared Spectroscopy. Front Neurosci 2020: 14: 300.

3. Toronov V, Webb A, Choi JH, Wolf M, Safonova L, Wolf U, Gratton E. Study of local cerebral hemodynamics by frequency-domain near-infrared spectroscopy and correlation with simultaneously acquired functional magnetic resonance imaging. Opt Express 2001: 9(8): 417-427.

4. Fantini S, Franceschini MA, Fishkin JB, Barbieri B, Gratton E. Quantitative determination of the absorption spectra of chromophores in strongly scattering media: a light-emitting-diode based technique. Appl Opt 1994: 33(22): 5204-5213.

5. Fantini S, Hueber D, Franceschini MA, Gratton E, Rosenfeld W, Stubblefield PG, Maulik D, Stankovic MR. Non-invasive optical monitoring of the newborn piglet brain using continuous-wave and frequency-domain spectroscopy. Phys Med Biol 1999: 44(6): 1543-1563.

6. Fantini S, Franceschini MA, Gratton E. Semi-infinite-geometry boundary problem for light migration in highly scattering media: a frequency-domain study in the diffusion approximation. Journal of the Optical Society of America B 1994: 11(10): 2128-2138.

7. Farzam P, Starkweather Z, Franceschini MA. Validation of a novel wearable, wireless technology to estimate oxygen levels and lactate threshold power in the exercising muscle. Physiological Reports 2018: 6(7): e13664.

8. Kleiser S, Nasseri N, Andresen B, Greisen G, Wolf M. Comparison of tissue oximeters on a liquid phantom with adjustable optical properties. Biomed Opt Express 2016: 7(8): 2973-2992.

9. Stankovic MR, Maulik D, Rosenfeld W, Stubblefield PG, Kofinas AD, Drexler S, Nair R, Franceschini MA, Hueber D, Gratton E, Fantini S. Real-time optical imaging of experimental brain ischemia and hemorrhage in neonatal piglets. J Perinat Med 1999: 27(4): 279-286.

10. Fantini S, Franceschini M, Gratton E, Hueber D, Rosenfeld W, Maulik D, Stubblefield P, Stankovic M. Non-invasive optical mapping of the piglet brain in real time. Opt Express 1999: 4(8): 308-314.

11. Hallacoglu B, Sassaroli A, Wysocki M, Guerrero-Berroa E, Schnaider Beeri M, Haroutunian V, Shaul M, Rosenberg IH, Troen AM, Fantini S. Absolute measurement of cerebral optical coefficients, hemoglobin concentration and oxygen saturation in old and young adults with near-infrared spectroscopy. J Biomed Opt 2012: 17(8): 081406-081401.

12. Zhang Z, Schneider M, Laures M, Qi M, Khatami R. The Comparisons of Cerebral Hemodynamics Induced by Obstructive Sleep Apnea with Arousal and Periodic Limb Movement with Arousal: A Pilot NIRS Study. Front Neurosci 2016: 10: 403.

13. Zhang Z, Khatami R. Predominant endothelial vasomotor activity during human sleep: a near-infrared spectroscopy study. Eur J Neurosci 2014: 40(9): 3396-3404.

14. Cleveland WS, Devlin SJ. Locally Weighted Regression - an Approach to Regression-Analysis by Local Fitting. J Am Stat Assoc 1988: 83(403): 596-610.

15. Quaresima V, Sacco S, Totaro R, Ferrari M. Noninvasive measurement of cerebral hemoglobin oxygen saturation using two near infrared spectroscopy approaches. J Biomed Opt 2000: 5(2): 201-205.

16. Scheeren TW, Schober P, Schwarte LA. Monitoring tissue oxygenation by near infrared spectroscopy (NIRS): background and current applications. J Clin Monit Comput 2012: 26(4): 279-287.

17. Al-Rawi PG, Kirkpatrick PJ. Tissue oxygen index: thresholds for cerebral ischemia using near-infrared spectroscopy. Stroke 2006: 37(11): 2720-2725.

18. Zhang Z, Khatami R. A Biphasic Change of Regional Blood Volume in the Frontal Cortex During Non-rapid Eye Movement Sleep: A Near-Infrared Spectroscopy Study. Sleep 2015.
